# From Surviving to Thriving: Key Considerations for Weight Control Across Diverse Cancer Survivorship Populations

**DOI:** 10.1002/osp4.70027

**Published:** 2024-11-29

**Authors:** Justin C. Brown, Marlyn Allicock, Carmina G. Valle, Tanya Agurs‐Collins

**Affiliations:** ^1^ Pennington Biomedical Research Center Baton Rouge Louisiana USA; ^2^ LSU Health Sciences Center New Orleans School of Medicine New Orleans Louisiana USA; ^3^ Stanley S. Scott Cancer Center Louisiana State University Health Sciences Center New Orleans Louisiana USA; ^4^ Louisiana Cancer Research Center New Orleans Louisiana USA; ^5^ Health Science Center at Houston School of Public Health Department of Health Promotion and Behavioral Sciences The University of Texas Dallas Texas USA; ^6^ Department of Nutrition Gillings School of Global Public Health University of North Carolina at Chapel Hill Chapel Hill North Carolina USA; ^7^ Lineberger Comprehensive Cancer Center University of North Carolina at Chapel Hill Chapel Hill North Carolina USA; ^8^ Division of Cancer Control and Population Sciences National Cancer Institute Rockville Maryland USA

**Keywords:** cancer survivors, obesity, prognosis, research gaps, weight management

## Abstract

**Background:**

Obesity is a chronic, relapsing, progressive disease of excess adiposity that increases the risk of dying from at least 16 types of cancer. The prevalence of obesity has increased more rapidly in cancer survivors compared with the general population. Tailored weight management strategies are needed to improve prognosis and health outcomes in the growing population of cancer survivors. However, certain cancer survivor population subgroups require unique consideration when developing weight management strategies.

**Methods:**

In a symposium convened by The Obesity Society during ObesityWeek 2023 titled “From Surviving to Thriving: Key Considerations for Weight Control Across Diverse Cancer Survivorship Populations,” experts presented the current state of the science and highlighted existing research gaps.

**Results:**

Topics included key considerations for weight management in adolescent and young adult cancer survivors, older adult cancer survivors, and understudied cancer survivor subgroups at high risk for poor health outcomes and innovative interventions that can be tested to improve cancer survivorship.

**Conclusions:**

This report reviews the symposium and offers perspectives from the expert panel about unique opportunities for future research on tailored weight management strategies to equitably improve prognosis and health outcomes in the diverse and growing population of cancer survivors.

## Graphical abstract

Without corrective action, the increasing burden of obesity in cancer survivors may impede progress in reducing death from cancer. Evidence‐based weight management strategies that are tailored to the unique needs of cancer survivorship subpopulations can promote equity in health outcomes and optimal survivorship after cancer for all.

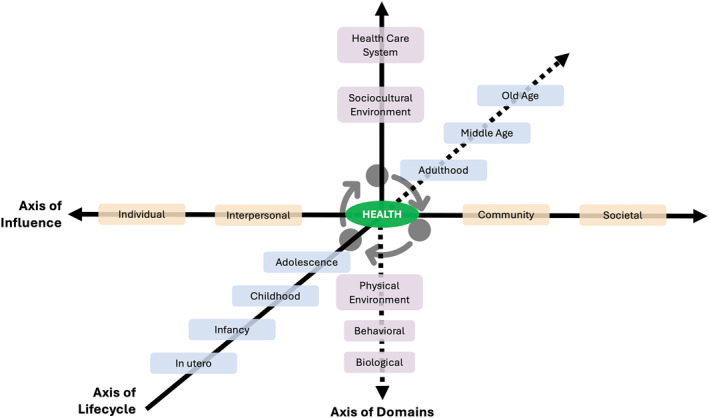

## 1 Introduction

One in 20 people in the U.S. is a cancer survivor [1]. The current population of 18.1 million cancer survivors in the U.S. is expected to grow to 26 million by 2040 [2]. The substantial growth of the cancer survivor population is attributed to improvements in early detection, advances in surgical, medical, and radiotherapy treatment techniques, and the success of population‐level behavioral health interventions (e.g., smoking cessation) [3]. Despite reductions in cancer death rates, not all cancer survivor subgroups have benefited equally [4]. Moreover, it is hypothesized that the pace of declining death rates has decelerated partly due to the increasing burden of obesity [5].

Obesity is a chronic, relapsing, progressive disease of excess adiposity. Obesity increases the risk of dying from at least 16 types of cancer [6]. The prevalence of obesity has increased more rapidly in cancer survivors compared with the general population [7]. Moreover, common adiposity‐based chronic diseases, such as type 2 diabetes and cardiovascular disease, may further increase cancer risk and worsen cancer outcomes [8]. The mechanisms hypothesized to underpin the association between obesity and cancer are likely multifactorial and have been detailed elsewhere [9].

Despite recommendations on nutrition, physical activity, and weight management from the American Cancer Society and other professional organizations [10], less than 5% of cancer survivors self‐report being fully adherent to such guidelines [11]. Emerging studies also report that cancer survivors experience weight stigma and bias in cancer‐related care [12]. Weight management strategies that are designed to address the unique needs of cancer survivors (e.g., tailored strategies) are needed to improve prognosis and health outcomes in the growing population of cancer survivors [13]. However, certain cancer survivor population subgroups require unique consideration when developing weight management strategies.

In a recent symposium convened by The Obesity Society and the Weight Management Dietetic Practice Group of the Academy of Nutrition and Dietetics during ObesityWeek 2023 titled “From Surviving to Thriving: Key Considerations for Weight Control Across Diverse Cancer Survivorship Populations,” experts presented the current state of the science and highlighted existing research gaps. Topics that were discussed included key considerations for weight management in (i) adolescent and young adult (AYA) cancer survivors, (ii) older adult cancer survivors, (iii) understudied cancer survivor subgroups at high risk for poor health outcomes (e.g., sexual and gender minorities, Hispanic & Latino, American Indian and Alaskan Native populations), and (iv) innovative interventions that can be tested to improve cancer survivorship equitably. This report aims to provide a concise overview of the symposium and offer perspectives from the expert panel about unique opportunities for future research; however, it is not intended to serve as a comprehensive review of all aspects of these topics.

### 1.1 Key Considerations for Adolescent and Young Adult Cancer Survivors

An estimated 86,000 AYAs, aged 15–39 years, are diagnosed with cancer each year in the United States, and cancer incidence rates have increased over the last decade, particularly among those aged 30–39 years, compared with a decreased incidence among those aged 50 and older [14]. Fortunately, overall death rates among AYAs have declined in recent decades [15]. As a result, many AYAs are living longer and may spend several decades grappling with the late and long‐term effects of cancer treatment and may be vulnerable to developing other chronic conditions. An estimated 30% of AYAs have obesity (National Health Interview Survey between calendar years 2013 and 2017) [16]. Additionally, AYAs with obesity have an increased risk of frailty or pre‐frailty [17, 18]. Some studies indicate that the prevalence of frailty in AYAs is comparable to that of 65‐year‐olds without cancer [17, 18]. Moreover, compared with those classified as robust, AYAs with frailty have elevated expression of p16, a biomarker of cellular senescence and biological aging, indicating a 35‐year acceleration of age [19].

AYAs may face unique challenges distinct from pediatric and older cancer survivors, including psychosocial concerns, distress, anxiety, and impacts on body and body image, as cancer interrupts an important period of development and growth, from adolescence through young adulthood, when individuals are developing their identity and independence [20, 21, 22]. Cancer may affect the remainder of an AYA's life, negatively impacting their health and well‐being over decades [23]. To date, none of these challenges are addressed in traditional weight management programs, and there is a need for weight management interventions specifically designed for and adapted to meet the needs of AYAs [24]. Behavioral weight loss interventions can safely promote clinically meaningful weight loss (i.e., 3%−5%) in cancer survivors, yet most of these studies have been among breast cancer survivors (mean age: 53.7) [25, 26]. A systematic review of 27 weight management interventions (including 16 randomized trials), including survivors of various cancer types, reported significantly decreased body weight in 22 out of the 27 interventions, yet no trials were focused on AYAs [27].

There are several key considerations to guide the design and evaluation of weight management interventions to address the needs of AYAs. It is critical to consider the unique challenges experienced by AYAs. While there is high interest among AYAs for weight management interventions and support to change their health behaviors [28], this is in the context of many significant life disruptions. Attending to the barriers (e.g., fatigue, depression, and treatment side effects) and facilitators (e.g., providing goals, offering social support, and implementing tailored programs) that AYAs have reported related to making healthy lifestyle changes after a cancer diagnosis may improve the uptake and efficacy of weight management interventions [29].

Common intervention preferences among AYAs include providing peer support and flexibility, enabling choice, and developing home‐based, tailored, and digital strategies [30, 31]. AYAs are digital natives, and using technology with AYAs holds the potential to offer an experience uniquely tailored to their individual needs. Recent reviews show that digital lifestyle interventions for AYAs may promote improvement in healthy behaviors, but most studies have been small pilot or short‐term feasibility trials [32, 33].

Engaging AYAs in adaptations of previous evidence‐based weight management programs is critical to systematically designing and tailoring effective interventions. To ensure that strategies are timely and relevant to their unique needs, AYAs can be engaged through community advisory boards and/or in co‐creating intervention approaches (e.g., dose, duration, features). The systematic use of established frameworks to guide intervention development and refinement [e.g., Multiphase Optimization Strategy (MOST), Behavior Change Wheel, or Obesity‐Related Behavioral Intervention Trials (ORBIT) models] [34, 35, 36] can advance the science and optimization of tailored weight management interventions for AYA cancer survivors.

Further advancement in weight management can be achieved through attention to both psychosocial and physiological mechanisms of action underlying interventions. Utilizing theoretical frameworks (e.g., self‐determination theory and social cognitive theory) [37], evidenced‐based behavior change techniques [27, 38], considering salient psychosocial determinants (e.g., stress and anxiety) [39, 40], and attending to the social drivers of health that are relevant to the unique needs and motivations of AYAs may improve the efficacy of weight management interventions [41]. Little is known about the physiological mechanisms of action that underlie the changes in body weight and body composition in AYAs, and the reasons for weight gain and fat mass accumulation are not well understood in cancer survivors broadly [42]. Attending to both psychosocial and physiological mediators of weight management through the systematic design of interventions and the evaluation of underlying mediators of interventions will enhance our ability to tailor future weight management interventions for cancer survivors.

Careful consideration should be given to outcome measures used in weight management trials in AYAs [13]. Patient‐reported outcomes particularly relevant to AYAs include psychological factors (e.g., anxiety, self‐esteem, and body image), social factors (e.g., relationships with peers), and treatment‐related symptoms (e.g., pain and fatigue), and possible adverse consequences of weight management interventions should be considered [13].

Given the tremendous heterogeneity within the AYA populations concerning cancer‐related characteristics, exercise preferences, and psychosocial functioning, heterogeneity in treatment response could be particularly salient [13, 43]. More research is needed to identify factors that may moderate intervention response (e.g., sociodemographic, cancer‐related, and social drivers of health) and advance our understanding of the AYAs who might most likely benefit from various intervention approaches. Considering a standard set of outcomes for consistency and comparison across weight management trials [e.g., use of the Accumulating Data to Optimally Predict obesity Treatment (ADOPT) Core Measures] could help advance our understanding of heterogeneity in response to treatments and explore physiological mechanisms of action [44].

### 1.2 Key Considerations for Older Adult Cancer Survivors

The life expectancy of older Americans continues to increase, with persons aged 65 years or older representing the fastest‐growing segment of the U.S. population [45]. The population of older adult cancer survivors is expected to increase from 62% to 73% over the next 2 decades [2]. The predicted demographic shift in the cancer survivor population will strain traditional healthcare models because many older adults often have age‐related (e.g., geriatric) syndromes that are present at cancer diagnosis [46], and because of the physiologic stress of cancer therapy, existing geriatric syndromes may be exacerbated, and additional syndromes are precipitated [47]. The interaction between older age, cancer, and cancer therapy culminates in a clinically relevant accelerated aging phenotype [48]. To address the unique needs of older adult cancer survivors, there are several key considerations to guide the design and evaluation of tailored weight management interventions.

Geriatric syndromes such as sarcopenia, frailty, falls, and mobility disability may worsen with cancer and cancer therapy. Sarcopenia is a syndrome of age‐related skeletal muscle mass loss that is common in cancer survivors and can be exacerbated by certain cancer therapies, such as the tyrosine kinase inhibitor sorafenib [49]. Falls are common in older adult cancer survivors, particularly among those with lower‐extremity chemotherapy‐induced peripheral neuropathy [50], and can cause bone fractures at rates higher than older adults without a history of cancer [51]. Frailty is a syndrome of poor global health and vulnerability to stressors that occurs in 42% of cancer survivors [52]. Although disability is distinct from frailty, disability can be a consequence of frailty [52]. In a nationally representative survey, one in three cancer survivors was classified as having a mobility disability characterized by poor self‐reported and objectively measured physical function [53].

These geriatric syndromes occur against the backdrop of obesity. Although the rates of obesity in older adults in the U.S. parallel that of younger generations [54], cancer survivors may be particularly vulnerable to developing obesity relative to their age and sex‐matched peers without cancer [7]. Consequently, geriatric syndromes and obesity often coexist in older adults; for example, obesity is associated with an increased risk of frailty [55], and frailty increases the risk of mortality [56]. The convergence of geriatric syndromes and obesity has created the opportunity to determine if weight management interventions can prevent the development or progression of various geriatric syndromes and improve health outcomes in older adult cancer survivors [57].

Observational studies in survivors of cancer report that weight loss is associated with an increased risk of death [58]. However, many observational studies cannot differentiate intentional weight loss (e.g., due to behavioral modification) from unintentional weight loss (e.g., due to underlying disease) as the mode of weight loss is prognostically distinct in humans [13]. For example, among community‐dwelling men, compared to those who were weight stable, men who experienced unintentional weight loss had a 71% higher relative risk of death [Hazard Ratio: 1.71 (95% Confidence Interval: 1.33, 2.19)], whereas men with intentional weight loss had a 41% lower relative risk of death [Hazard Ratio: 0.59 (95% Confidence Interval: 0.34, 1.00)] [59]. This is consistent with meta‐analyses of dietary intervention weight loss trials in healthy adults, which reported that randomization to a weight‐reducing diet was associated with a statistically significant 18% lower relative risk of all‐cause mortality than control [60].

Most cancer survivors are older adults, yet older adults are underrepresented in oncology clinical trials [61]. Several trials have exclusively enrolled older adult cancer survivors, which have provided foundational discoveries to advance the care of older adult cancer survivors and inform the future research agenda. For example, Project LEAD was a trial of 182 survivors of breast and prostate cancer who were aged ≥ 65 years or older and randomized to a home‐based diet and exercise program or an attention control condition [62]. Although diet quality was statistically significantly improved in the diet and exercise program at week 24, physical activity energy expenditure and physical functioning (primary endpoint) were not improved [63]. The RENEW trial was a trial of 641 older adult survivors of breast, colorectal, and prostate cancer with overweight or obesity who were randomized to a home‐based lifestyle program of diet, exercise, and modest weight loss or waitlist control. The lifestyle program attenuated the loss of self‐reported physical functioning at week 52, and this effect persisted at week 104 [64, 65]. These studies support the premise that behavioral changes to improve diet quality, increase physical activity, and induce modest weight loss are feasible and produce clinically valuable improvements in health in older adult cancer survivors.

The co‐existence and synergy between geriatric syndromes common in older adult cancer survivors and obesity will strain our healthcare system. However, this has created an opportunity to determine if tailored weight management strategies prevent the development and progression of geriatric syndromes, when such strategies should be deployed along the cancer control continuum, and to whom they should be delivered.

### 1.3 Key Considerations for Population Subgroups at Risk for Health Disparities

Racial minorities in the U.S. experience a disproportionate burden of illness and disease that is reflected in racial health disparities. Substantial disparities in the prevalence of overweight and obesity occur across racial, ethnic, and socioeconomic status (SES) [66, 67]. The prevalence of overweight and obesity is higher among women, racial and ethnic minorities, and those from lower SES [68, 69]. Tailored weight management interventions to address cancer disparities should recognize the racial and ethnic differences in biological, behavioral, and cultural attributes. Race and ethnicity shape life contexts and impact factors related to diet and physical activity that may differ across race and ethnic populations [70]. Given that the majority of research to date has been conducted in primarily non‐Hispanic white populations, it is imperative to understand these influences to intervene effectively and improve health outcomes.

#### 1.3.1 Non‐Hispanic Black Population

Systemic racism is purported to be a main driver of the disproportionate gaps in mortality and incidence for the non‐Hispanic black (NHB) population, which is reflected in the unequal distribution of poverty and reduced access to equitable healthcare [71]. NHB men (along with American Indian or Alaska Native men) have the highest overall cancer mortality (≈19% higher than White men), and NHB women have 40% higher breast cancer mortality than White women despite having lower incidence rates [71]. The NHB population in the US has the highest prevalence of obesity among all racial and ethnic subgroups [72]. Physical inactivity and excessive weight gain that begins in childhood and persists into adulthood results in long‐term cumulative excess body fat, negatively impacting health [73]. Cultural differences and preferences in personal body image are other key factors that may mediate obesity outcomes; for example, the NHB population is more likely to underestimate their weight status [74]. Barriers to engaging in physical activity among NHB cancer survivors include lack of energy, time, limited access to appropriate facilities, and lack of knowledge about activity [75]. Additionally, the high prevalence of limited access to healthy and affordable foods and the low prevalence of safe spaces for physical activity means that the NHB population has more challenges consuming healthy foods and engaging in sufficient physical activity, both of which contribute to obesity risk [76].

#### 1.3.2 LGBTQ+ Population

Excess body weight is a risk factor for developing various cancers among U.S. adults who identify as lesbian, gay, bisexual, transgender, queer, questioning, or other diverse sexual orientation or gender identity (LGBTQ+)—referred to as sexual and gender minorities. Lesbian and bisexual women are likely to have excess body weight compared to heterosexual women [77, 78], whereas others found that gay males have lower odds of being overweight compared to heterosexual men [77, 78]. Factors that contribute to the increased risk of obesity may relate to lower physical activity levels, stress, eating disorders, body shaming, and the influence of pervasive sexual objectification of the female body [10, 79]. Studies have consistently shown that gay men tend to have higher rates of eating disorders (e.g., less control overeating, food addiction, and unhealthy weight control behaviors) [80, 81, 82]. Studies have also reported a greater prevalence of overweight and obesity among sexual and gender minority adolescents compared with cisgender, heterosexual youth [83, 84, 85, 86]. There is a more limited body of research regarding sexual and gender minority cancer survivors with obesity, although they experience significant cancer disparities [87, 88, 89]. With respect to obesity and obesity‐risk behaviors, some studies have found that lesbian female cancer survivors have higher odds of being overweight or obese compared to their peers without cancer [90]. Gay male cancer survivors engage in lower amounts of physical activity compared with heterosexual male cancer survivors [91]. Cancer health disparities among sexual gender minority groups is a recognized research priority [92, 93, 94], where current knowledge and gaps remain concerning obesity experiences, care, and outcomes. The need for research is to (1) examine sexual minority subgroups [77] and (2) focus on the intersecting marginalized identified among sexual gender minority people [95].

#### 1.3.3 Hispanic and Latino Population

The Hispanic population comprises more than 20 heritage groups and is the second‐largest racial and ethnic group, representing 19% of the U.S. population [71]. Despite the higher rates of obesity and the relatively high rates of some obesity‐related cancers, Hispanic populations have lower overall cancer incidence and cancer‐specific mortality [96]. Cancer is one of the leading causes of death, accounting for 20% of deaths among Hispanic and Latino adults [97]. Even with having lower overall cancer rates than the non‐Hispanic White population, cancers of the stomach, liver, and cervix are more prevalent among Hispanic and Latino individuals [97]. Additionally, Hispanic and Latino adults have one of the highest rates of excess body weight, with 44.8% having obesity and 60.6% having abdominal obesity (defined using waist circumference), particularly among women [98]. Hispanic women remain underrepresented in weight loss research, including a systemic review highlighting the paucity of randomized trials focused on diet and physical activity to promote weight loss for Hispanic women [99]. Diet quality, sedentary behaviors, and low physical activity are known risk factors for obesity among the Hispanic and Latino populations [100]. However, the compounding effects of multiple social determinants, such as low SES, structural racism, limited access to quality evidence‐based cancer care, lack of trust in healthcare providers, and patient‐provider communication challenges, further contribute to the health disparities experienced by the Hispanic and Latino populations [97, 101, 102].

#### 1.3.4 American Indian or Alaska Native Population

The American Indian or Alaska Native (AIAN) population, which represents about 3% of the U.S. population, has a higher prevalence of many chronic health conditions than any other racial or ethnic group in the U.S. [103]. Lung, colorectal, kidney, liver, stomach, and cervical cancer incidence is higher in the AIAN population than in the non‐Hispanic White population [104]. Later‐stage diagnosis and poorer overall survival among the AIAN population are common, with marked disparities in breast and stomach cancers, which are attributed to reduced access to high‐quality care and barriers to early detection and treatment [105]. Compared to the non‐Hispanic White population, AIAN individuals are more likely to have obesity (40% vs. 31%). Food insecurity and low access to healthy foods attributed to the disproportionate poverty and rural environment of the AIAN population are often cited as primary contributors to obesity [106]. Efforts to address limited access to grocery stores (mainly the Food Distribution Program on Indian Reservations) have exacerbated this problem, in part, by supplying highly processed, high‐calorie foods. Although the traditional native American diet includes frequent consumption of fruits and vegetables, which are associated with reduced obesity risk, historical injustices and racism exist [107]. A solution is to develop food sovereignty and expand access to locally grown foods to improve nutrition and reduce obesity among the AIAN population.

Tailored weight management interventions delivered in primary care settings among low‐income groups using individual‐level interventions are effective in the short term (up to 9 months) [108]. Similarly, community‐based behavioral weight management interventions have short‐term effectiveness. A research gap to explore further is quantifying differences in adiposity, skeletal muscle, and metabolic rate at the same body mass index across racial and ethnic groups, which may help understand disparities in obesity [109]. Others have also indicated that often unexamined social determinants related to minority status, such as exposure to racism and the impact of racism (e.g., racial discrimination, residential segregation, and challenges to accumulating wealth), such as denying certain minority groups the opportunity for social and economic growth, sufficient capital, and adequate to high‐quality healthcare, may contribute to creating disparities to an extent that has been previously realized [110].

### 1.4 Pressing Questions to Advance the Field

The panel discussed several opportunities for future research, including (i) better utilization of existing theoretical models to inform intervention development and study design, (ii) deployment of multilevel interventions that systematically and simultaneously augment multiple factors that contribute to health, and (iii) the thoughtful integration of technology to equitably enhance reach and efficacy. Embracing these opportunities for future research may enable cancer survivor populations across the lifespan to derive the health benefits of tailored weight management interventions.

The success of multimodal behavioral weight management interventions that consist of calorie restriction, physical activity, and behavioral counseling in cancer survivors is improved when coupled with theoretical models. Theoretical models help guide study development and study design and can be used to systematically identify factors that influence intervention effectiveness. Several systematic reviews and meta‐analyses support the utilization of theory‐based interventions for diet and physical activity behavior change among cancer survivors; however, there are important challenges [111, 112]. Although these models show promise, many do not consider environmental and societal influences that are known to impact health behaviors [113].

The socio‐ecological model considers both higher‐level environmental or upstream factors, such as organizational‐ or policy‐level factors, and individual biological or downstream factors that contribute to health [114]. The socio‐ecological model postulates that individuals are embedded within a more extensive social system, and the model attempts to describe the interactions between individuals and their environment that influence health outcomes [115].

**FIGURE 1:**
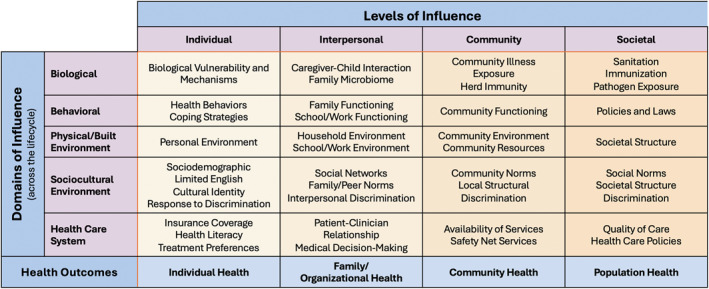
National Institute of Minority Health and Health Disparities Research Framework.

The National Institute on Minority Health and Health Disparities (NIMHD) research framework expands on the socio‐ecological model to help explain the different determinants contributing to health disparities. The NIMHD framework (Figure 1) is a multilevel and multidomain model that depicts an array of health determinants relevant to minority health and health disparities that span multiple domains of influence (e.g., biological, behavioral, the physical and built environment, sociocultural environment, health care system) and levels of influence on health (e.g., individual, interpersonal, community, and societal) [116]. This framework can guide the development of cancer prevention and control interventions, particularly in populations that experience health disparities.

A meta‐analysis of weight gain after breast cancer diagnosis observed a higher risk of all‐cause mortality among patients who gained weight compared with patients who remained weight stable [117]. Many factors are associated with weight gain after a breast cancer diagnosis. For example, a prospective study of non‐Hispanic Black breast cancer survivors identified several individual and neighborhood factors associated with post‐diagnosis adiposity changes, including premenopausal status, treatment with chemotherapy, decreased physical activity, and residence in neighborhoods with a higher density of fast‐food‐style restaurants [118]. Applying the NIMHD framework, a weight maintenance intervention could be designed to improve diet quality (behavioral domain at the individual level), develop culturally appropriate community physical activity programs (sociocultural domain at the community level), and promote or enforce policies (e.g., zoning laws) to increase access to healthy foods (physical environment at the societal level) after a breast cancer diagnosis.

**FIGURE 2:**
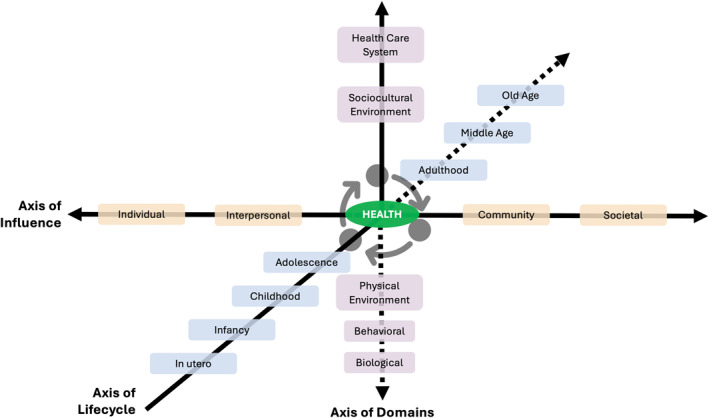
A conceptual model that outlines how the lifecycle intersects domains and levels of influence that interact to produce health outcomes at the individual, family, community, and population levels—adapted from the National Institute of Minority Health and Health Disparities Research Framework.

We have adapted the NIMHD framework to explicitly acknowledge how the lifecycle intersects domains and levels of influence that interact to produce health outcomes at various levels (Figure 2). This framework can inform the design of multilevel interventions to address the determinants that influence obesity risk and adverse health outcomes among racial and ethnic minority groups or other populations across the lifespan.

Multilevel interventions may be more effective than single‐level interventions because they specifically target two or more levels of influence and can leverage interactions between levels that create synergistic intervention effects [119]. For example, a systematic review of multilevel interventions to decrease sedentary behavior in children found that interventions were more effective when they targeted four levels of influence: intrapersonal, interpersonal, organizational, and community [120].

Technology (e.g., internet‐based [electronic health, eHealth] technologies, mobile [mobile health, mHealth] apps, and telemedicine) may improve access to care and empower patients to participate actively in their care, providing a means to reduce healthcare disparities [121]. For example, automated online behavioral obesity treatment and maintenance interventions can induce weight loss at 12 and 24 months [122]. Although technology preferences to promote behavior change may vary, cell phone, intervention, and social media use are common across many population subgroups [123]. Nonetheless, as technology‐based interventions are tested, attention should be paid to common barriers such as lack of video sharing capabilities, inconsistent internet availability or internet access, lack of private spaces to participate in virtual visits, and language barriers.

## 2 Conclusion

Without corrective action, the increasing burden of obesity in cancer survivors is likely to impede progress in reducing death from cancer. Tailored weight management interventions are critical to equitably improve prognosis and health outcomes in the growing population of cancer survivors. However, certain cancer survivor population subgroups, such as AYAs, older adults, and those vulnerable to health disparities, require unique consideration when developing weight management interventions. The development and implementation of innovative, evidence‐based weight management interventions that are tailored to the unique needs of cancer survivorship subpopulations will equitably improve prognosis and health outcomes in the diverse and growing population of cancer survivors.

## Author Contributions

Conceptualization: J.C.B., M.A., C.G.V., T.A.‐C.; Data curation: not applicable; Formal analysis: not applicable; Funding acquisition: not applicable; Investigation: J.C.B., M.A., C.G.V., T.A.‐C.; Methodology: J.C.B., M.A., C.G.V., T.A.‐C.; Project administration: J.C.B., M.A., C.G.V., T.A.‐C.; Resources: not applicable; Software: not applicable; Supervision: J.C.B., T.A.‐C.; Validation: J.C.B., M.A., C.G.V., T.A.‐C.; Visualization: J.C.B., M.A., C.G.V., T.A.‐C.; Writing – original draft: J.C.B., M.A., C.G.V., T.A.‐C.; Writing ‐ review & editing: J.C.B., M.A., C.G.V., T.A.‐C.; all authors had final responsibility for the decision to submit for publication.

## Conflicts of Interest

Dr. Brown reports receiving grants from the National Institutes of Health, the American Institute for Cancer Research, and Cancer Research UK that were paid to his institution. Dr. Brown also reports receiving consulting fees from Novo Nordisk A/S and Nestlé Health Sciences. All other authors disclosed no conflicts of interest. The research suggestions in this manuscript should not be viewed as an indication of National Cancer Institute research priorities.
